# Functional Specialization within the EXO70 Gene Family in Arabidopsis

**DOI:** 10.3390/ijms22147595

**Published:** 2021-07-15

**Authors:** Vedrana Marković, Ivan Kulich, Viktor Žárský

**Affiliations:** 1 Department of Experimental Plant Biology, Faculty of Science, Charles University, Viničná 5, 12844 Prague, Czech Republic; vedrana.markovic@natur.cuni.cz (V.M.); viktor.zarsky@natur.cuni.cz (V.Ž.); 2 Institute of Experimental Botany, Czech Academy of Sciences, Rozvojová 263, 16502 Prague, Czech Republic

**Keywords:** exocyst complex, EXO70, Arabidopsis, EXO70A1, polar exocytosis, EXO70B1

## Abstract

Localized delivery of plasma-membrane and cell-wall components is a crucial process for plant cell growth. One of the regulators of secretory-vesicle targeting is the exocyst tethering complex. The exocyst mediates first interaction between transport vesicles and the target membrane before their fusion is performed by SNARE proteins. In land plants, genes encoding the EXO70 exocyst subunit underwent an extreme proliferation with 23 paralogs present in the Arabidopsis (*Arabidopsis thaliana*) genome. These paralogs often acquired specialized functions during evolution. Here, we analyzed functional divergence of selected EXO70 paralogs in Arabidopsis. Performing a systematic cross-complementation analysis of *exo70a1* and *exo70b1* mutants, we found that EXO70A1 was functionally substituted only by its closest paralog, EXO70A2. In contrast, none of the EXO70 isoforms tested were able to substitute EXO70B1, including its closest relative, EXO70B2, pointing to a unique function of this isoform. The presented results document a high degree of functional specialization within the EXO70 gene family in land plants.

## 1. Introduction

Exocytosis is an essential process in all eukaryotic cells when secretory vesicles fuse with the plasma membrane (PM) and deliver various cargo to the PM or extracellular space. In eukaryotic organisms with cell walls, the exocytosis underlies their cell morphogenesis because it plays a crucial role in assembly of the cell wall by delivering some of its components. The delivery of such cargo in secretory vesicles is often restricted to specific sites at the PM resulting in polarized secretion. One of the fundamental regulators of polarized secretion is the exocyst tethering complex [1,2,3]. The exocyst localizes to sites of intensive secretion where it facilitates an initial interaction between the secretory vesicle and plasma membrane prior to the final SNARE-mediated vesicle–membrane fusion. Controlled by small GTPases, the exocyst provides the spatio-temporal specificity to the targeting of secretory vesicles. The exocyst is an evolutionary-conserved protein complex which consists of eight subunits—Sec3, Sec5, Sec6, Sec8, Sec10, Sec15, Exo70, and Exo84 [2,3]. In yeast and animals, each exocyst subunit is typically encoded by a single gene, but in land plants, exocyst genes are usually duplicated or even multiplied [4,5]. Especially, the *EXO70* gene underwent an extreme proliferation, generating 13 EXO70 paralogs in the genome of *Physcomitrella patens*, 47 paralogs in *Oryza sativa*, and 23 paralogs in *Arabidopsis thaliana* [5].

The EXO70 gene family of land plants originated from three ancient EXO70 paralogs and can be classified into three ancestral subfamilies—EXO70.1, EXO70.2, and EXO70.3 [5]. The EXO70.1 subfamily, consisting exclusively of EXO70A isoforms, is engaged in the canonical exocyst function in polarized exocytosis which is important for polar growth and cell-wall biogenesis. In Arabidopsis, EXO70.1 comprises three members—EXO70A1, EXO70A2, and EXO70A3. The best-described member of the EXO70A clade is EXO70A1 that localizes to the PM domains with high secretion (e.g., outer lateral PM of root epidermal cells) [6,7,8]. As shown previously, *exo70a1* mutant plants exhibit pleiotropic morphological defects such as loss of apical dominance, dwarfish appearance, and sterility [9]. Moreover, *exo70a1* mutants exhibit decreased PIN transporter recycling [10,11], slower root-cell elongation [12], compromised root hair growth [9,13], defective Casparian strips [14], and a thinner and collapsed xylem secondary cell wall [15,16]. Analogous to EXO70A1 in the sporophyte, its closest paralog, EXO70A2, is engaged in the regulation of exocytosis in the male gametophyte [17,18]. Mutants in EXO70A2 are characterized by defects in pollen grain maturation, germination, and pollen tube growth [17,18]. The *EXO70A3* gene plays a putative role in modulation of auxin-controlled root development by acting on the PIN4 distribution [19].

In contrast, the EXO70.2 subfamily, which is the most evolutionary dynamic, comprises six clades, namely, EXO70B, C, D, E, F, and H, which are involved in non-canonical exocyst functions or act independently of the exocyst complex [6]. The most-studied clade is EX070B comprising two paralogs in Arabidopsis. EXO70B1 plays a role in autophagy-related transport to the vacuole, stomatal regulation, and innate immunity [20,21,22]. Mutants lacking EXO70B1 accumulate decreased amounts of autophagic bodies in the vacuole, are hypersensitive to nitrogen starvation, and show compromised accumulation of anthocyanins [20]. EXO70B1, as a part of an exocyst subcomplex containing EXO84b and SEC5a, colocalized with anthocyanins and autophagy marker ATG8f inside the vacuole [20]. EXO70B2 acts in regulation of innate immunity. Mutants in EXO70B2 showed abnormal papillae formation upon *Blumeria graminis* infection and enhanced susceptibility to different elicitors and pathogens [23,24]. Two closely related members of the EXO70C clade, EXO70C1 and EXO70C2, expressed in root trichoblast cells and in pollen, typically show cytoplasmic localization and negatively regulate polar growth of pollen tubes independently of the exocyst complex [25]. A recent study demonstrated that members of the EXO70D clade mediate selective autophagic degradation of type-A ARR proteins to regulate cytokinin sensitivity [26]. A member of the EXO70E clade, EXO70E2, was found in double-membrane structures resembling autophagosomes that were named as *EXPOs* (exocyst-positive organelles) and proposed to be exported out of the cell in an exosome-like manner [27,28]. The EXO70F clade is still awaiting its detailed characterization. However, this clade underwent massive evolutionary expansion in monocots, where it is involved in plant defense [29,30]. The best-characterized member of the EXO70H clade is EXO70H4 that regulates polarized callose deposition during secondary thickening of the cell wall in Arabidopsis trichomes [31,32]. EXO70H1 has a role in plant defense. Plants lacking EXO70H1 exhibit enhanced susceptibility towards *Pseudomonas syringae* [23].

The third subfamily, EXO70.3, comprises EXO70G and EXO70I clades, with EXO70I being lost in Brassicaceae. So far, only the EXO70I clade is partly characterized in *Medicago trunculata*, where it is involved in the formation of the periarbuscular membrane subdomain during arbuscular mycorrhizal symbiosis [33].

The high number and diversity of plant-specific EXO70 paralogs, sharing 26% to 72% identity at the protein level, points to their functional specialization [6,25]. While EXO70C1 and EXO70C2 are highly redundant [25], EXO70H4 could not be substituted by any other EXO70 tested [32]. Double mutants *exo70a1*/*exo70b1* exhibited additive phenotypes of the respective single mutants, indicating a minimal functional overlap of these two isoforms [20]. Here, we provide further evidence of the functional divergence between EXO70 isoforms in Arabidopsis by performing a systematic cross-complementation analysis of *exo70a1* and *exo70b1* mutants with representatives of most clades of the three EXO70 subfamilies.

## 2. Results

### 2.1. EXO70A1 Can Be Functionally Substituted Only by EXO70A2 in Arabidopsis

**Figure 1:**
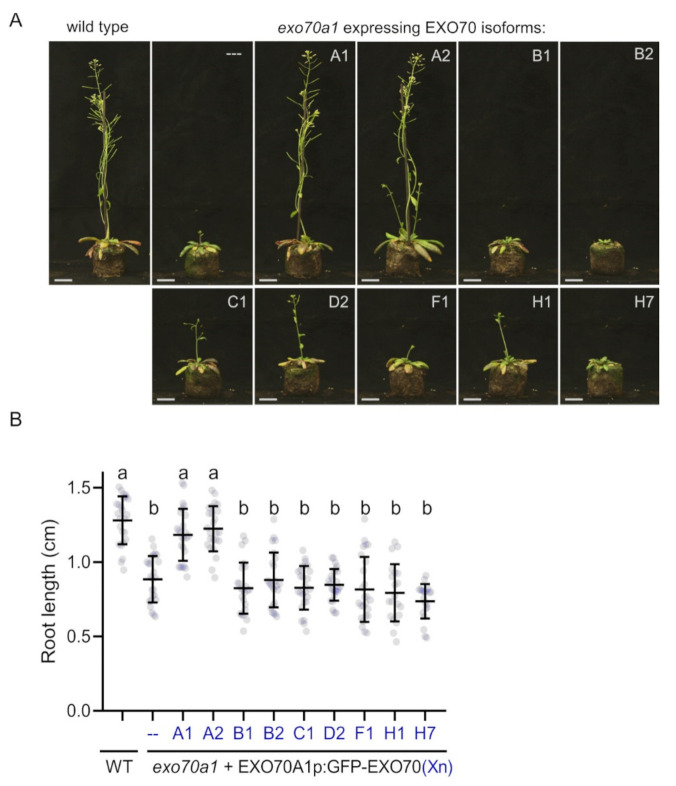
Complementation analysis of *exo70a1* mutant plants expressing different EXO70 isoforms; (**A**) representative images of 4-week-old plants expressing indicated GFP-tagged EXO70 isoforms under the control of *EXO70A1* promoter. WT and *exo70a1* are displayed as controls. At least two independent lines for each genotype were imaged. Scale bars = 2 cm. (**B**) Measurement of primary root lengths in 5-day-old seedlings of WT and *exo70a1* mutants. A1 to H7 indicate GFP-tagged EXO70 isoforms expressed in *exo70a1* mutants under the control of *EXO70A1* promoter. Primary roots of 20 plants per genotype were used for quantification (*n* = 20). Bold, horizontal lines represent the means, whereas error bars represent SD. Letters a and b denote statistically different groups calculated by one-way ANOVA with post hoc Tukey’s honest significance difference test; *p* < 0.01.

To investigate the functional relationship of EXO70A1 to other EXO70 isoforms in Arabidopsis, we performed a systematic cross-complementation analysis using the *exo70a1* knock-out mutant. We subcloned representatives from most EXO70 clades that are significantly expressed in roots (EXO70A1, A2, B1, B2, C1, D2, F1, H1 and H7), generating a series of constructs for stable expression of these EXO70s under control of the *EXO70A1* promoter and tagged with GFP on the N-terminus (EXO70A1p::GFP:EXO70). To overcome the *exo70a1* mutant sterility [9], the constructs were transformed into *exo70a1* heterozygotes. Then, we assessed the capacity of each EXO70 to complement the loss of the EXO70A1 function in homozygous *exo70a1* mutants segregating in the T2 generation. We found that unlike EXO70A2 and EXO70A1 itself, no other EXO70 tested was able to rescue the morphological defects of *exo70a1*, as demonstrated by general morphology of mature plants in soil (Figure 1A) and by measurement of primary root length in young seedlings in vitro (Figure 1B). Additionally, only complementation by EXO70A1 and EXO70A2 restored *exo70a1* mutant sterility.

**Figure 2:**
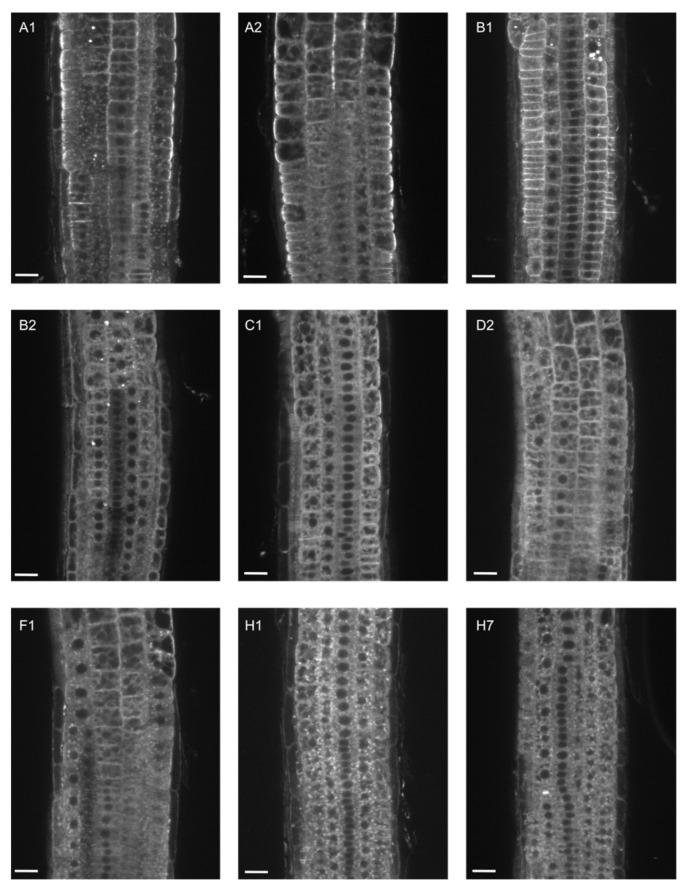
Localization of different EXO70 isoforms in the wild-type background. Representative images of 7-day-old seedlings expressing GFP-tagged EXO70 isoforms under the control of the *EXO70A1* promoter in wild-type background. Root regions from the apical to the basal meristematic zone were imaged consistently. At least two independent lines for each genotype were imaged. Scale bars = 20 µm.

**Figure 3:**
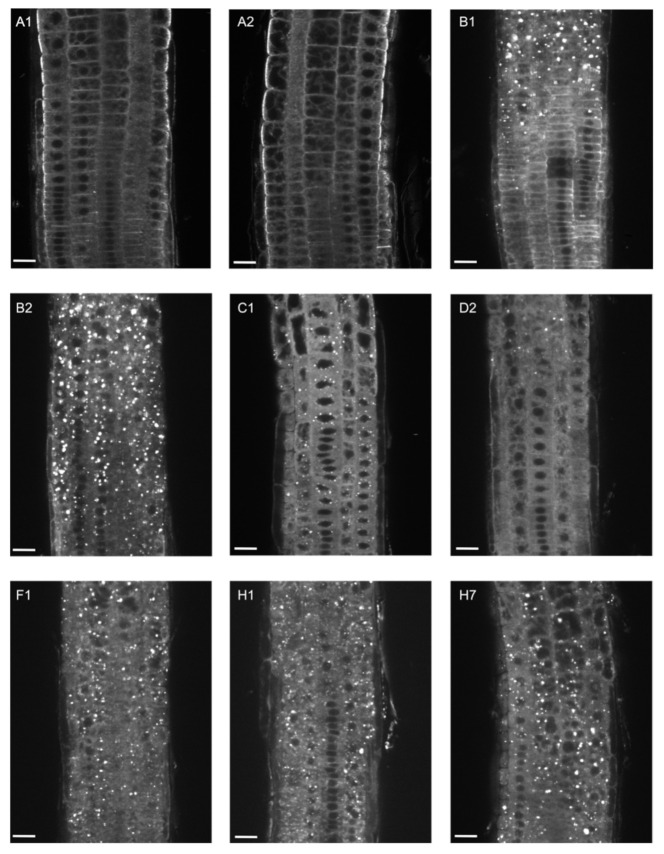
Localization of different EXO70 isoforms in the *exo70a1* mutant background. Representative images of 7-day-old seedlings expressing GFP-tagged EXO70 isoforms under the control of the *EXO70A1* promoter in the *exo70a1* background. Root regions from the apical to the basal meristematic zone were imaged consistently. At least two independent lines for each genotype were imaged. Scale bars = 20 µm.

Next, we analyzed GFP-EXO70 localization in root epidermal cells, where the *EXO70A1* promoter is predominantly active. We compared localization of each paralog in 5-day-old homozygous *exo70a1* mutant plants and in wild-type siblings (control) (Figure 2 and Figure 3).

In root epidermal cells of both *exo70a1* and WT plants, EXO70A2 localized identically to EXO70A1—partly in the cytoplasm and predominantly at the PM, with a typical enrichment at the outer lateral domain (Figure 2 and Figure 3). In WT plants, EXO70B1 showed a strong signal along the entire PM, but without the enrichment at the outer-lateral PM domain, while EXO70B2, C1, D2, F1, H1, and H7 accumulated mostly in the cytoplasm with a weak distribution along the PM (Figure 2).

However, in the *exo70a1* background, these isoforms, including EXO70B1, were localized in distinct intracellular aggregates (Figure 3) and the PM localization was almost completely lost as shown by quantification of the PM-to-cytoplasm GFP-fluorescence ratio (Supplemental Figure S1). Interestingly, these structures appeared in basal and apical root meristematic zone for EXO70B2, C1, F1, H1, and H7, but were much more abundant in the basal meristematic zone for EXO70B1 and D2 (Figure 3). Using membrane staining with the FM4-64 dye, we revealed that the structures represent a sort of endomembrane compartments (Supplemental Figure S2).

We conclude that among the EXO70 isoforms tested, only EXO70A2 has a potential to functionally substitute for EXO70A1 in the sporophyte.

### 2.2. EXO70B1 Is a Highly Specialized EXO70 Isoform with No Functionally Redundant Paralogs

**Figure 4:**
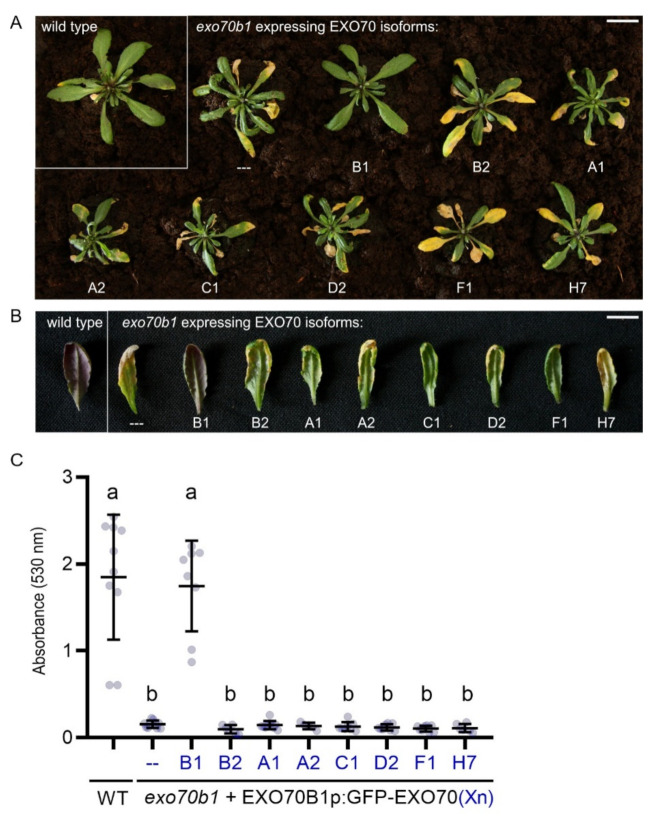
Complementation of *exo70b1* mutant plants by different EXO70 isoforms; (**A**) one-month-old Arabidopsis plants of wild type and *exo70b1*, which express indicated GFP-tagged EXO70 isoforms under control of the *EXO70B1* promoter. EXO70B1 was the only EXO70 isoform able to complement the early senescence phenotype of *exo70b1*. At least two independent lines for each genotype were imaged. Scale bar = 2 cm; (**B**) EXO70B1 is the only EXO70 isoform able to complement the anthocyanin accumulation defect in the *exo70b1* mutant. Scale bar = 2 cm; (**C**) quantification of absorbance by the pigments extracted from leaves in (**B**); leaves (20 mg per plant) collected from at least five plants (*n* > 5) per genotype were used for quantification; bold, horizontal lines represent the means, whereas error bars represent SD.; letters denote statistically different groups calculated by one-way ANOVA with post hoc Tukey’s honest significant difference test; *p* < 0.01.

**Figure 5:**
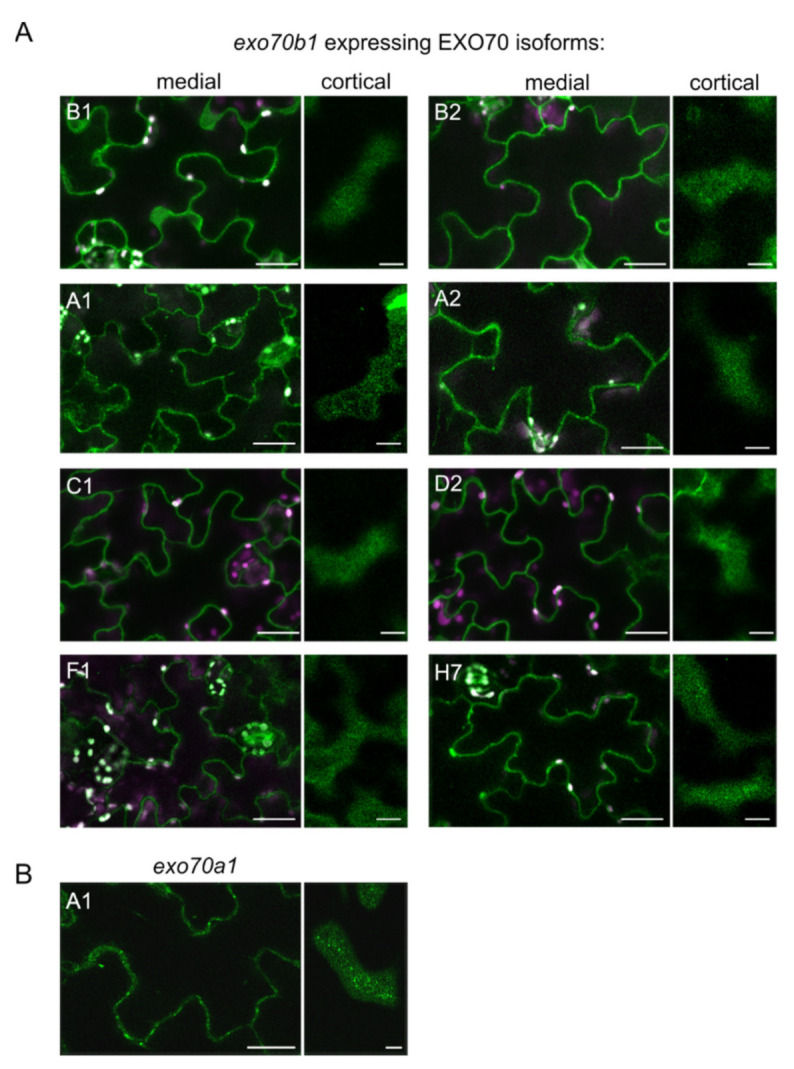
Localization of different EXO70 isoforms in abaxial epidermis in *exo70b1* mutant cotyledons; (**A**) representative images of epidermal cells in 7-day-old cotyledons expressing GFP-tagged EXO70 isoforms under control of the *EXO70B1* promoter in the *exo70b1* background. Cells were imaged in their medial and cortical planes; the cortical plane includes the PM. At least two independent lines for each genotype were imaged; (**B**) representative image of epidermal cells expressing *GFP:EXO70A1* under control of the *EXO70A1* promoter in the *exo70a1* background used as a positive control. Scale bars for medial and cortical sections are 20 µm and 10 µm, respectively. Magenta channel represents chlorophyll autofluorescence.

To explore the functional specificity of EXO70B1, we performed the cross-complementation analysis as above for EXO70A1. In this case, representatives from other EXO70 clades (EXO70A1, A2, B1, B2, C1, D2, F1, and H7) were expressed from the *EXO70B1* promoter with N-terminal GFP fusion. Generated constructs (EXO70B1p::GFP:EXO70) were then transformed into homozygous *exo70b1* mutants. Subsequently, we observed whether expressed EXO70 paralogs were able to rescue the early senescence and anthocyanin accumulation defect of the *exo70b1* mutant [20] (Figure 4). We also analyzed their localization patterns in cotyledons, where *EXO70B1* promoter is predominantly active (Figure 5).

First, we performed a phenotypic analysis of 1-month-old plants, because at this point *exo70b1* mutants develop the early senescence phenotype characterized by the presence of small spontaneous leaf lesions [20]. Among the EXO70s tested, only EXO70B1 itself was able to rescue the early senescence phenotype of *exo70b1* (Figure 4A). Second, we induced anthocyanin synthesis in 1-month-old plants, extracted leaf pigments after another month and assessed the content of anthocyanins. Apart from EXO70B1, no other EXO70 paralog could restore the anthocyanin accumulation defect in the *exo70b1* mutant (Figure 4B,C), suggesting that the function of EXO70B1 is highly specific for this process.

Finally, we investigated the localization patterns of GFP-tagged EXO70 isoforms under control of the *EXO70B1* promoter in cotyledons of 7-day-old *exo70b1* mutant seedlings (Figure 5). EXO70C1 and EXO70D2 localized predominantly in the cytoplasm in abaxial epidermal cells, however due to the strong cytoplasmic signal we cannot exclude PM localization (Figure 5A). All other EXO70 isoforms tested localized partly to the cytoplasm and significantly at the PM to distinct foci (Figure 5A). This localization pattern was comparable to that of EXO70A1p::GFP:EXO70A1 (in the *exo70a1* mutant background), which represents a typical pattern of exocyst subunits [7,17].

In conclusion, our data showed that EXO70B1 acquired a unique function within the EXO70 family.

## 3. Discussion

The interesting aspect of the plant exocyst is the enormous evolutionary proliferation of the gene family encoding the EXO70 subunit. The increased number of EXO70 paralogs in plants and their differential expression opens an important question of their functional redundancy. Here, we focused on the functional redundancy of EXO70A1 and EXO70B1, respectively, with their paralogs from other EXO70 clades.

We documented that, among all EXO70 isoforms tested, only EXO70A2 has the ability to functionally substitute EXO70A1 in the sporophyte. This agrees with the observation that the EXO70A2 disruption increased the effect of EXO70A1 disruption, because seedlings of the *exo70a1*/*exo70a2* double-mutant showed more severe phenotypes than the *exo70a1* mutant alone [17]. These two paralogs belong to the EXO70.1 subfamily and share 72% sequence identity at the protein level, documenting their close evolutionary relationship. In contrast, EXO70B1, B2, C1, D2, F1, H1, and H7 belong to the EXO70.2 subfamily and share only 22% to 32% identity with EXO70A1 [25]. This observation supports our notion that EXO70 paralogs from EXO70.2 and EXO70.3 subfamilies gained specific functions diverged from the EXO70.1 subfamily (i.e., EXO70A clade). Interestingly, when expressed in pollen, EXO70A1 was unable to replace EXO70A2 function, possibly due to its inability to bind EXO70A2-specific protein interactors present in the male gametophyte [17].

EXO70B1, B2, C1, D2, F1, H1 and H7 developed abnormal endomembrane compartments in root cells, when expressed in *exo70a1* mutant background. This indicates that in root cells PM targeting of different EXO70 subunits is dependent on EXO70A1. We speculate that EXO70 subunits might represent a cargo for EXO70A1-containing exocyst complex. However, it seems that the dependence on EXO70A1 is cell-specific since EXO70B1 expressed in *exo70a1* mutant retained the ability to bind the PM in the apical meristematic root zone. Core exocyst subunits also aggregated in those structures, indicating the importance of EXO70A1 for the recruitment of the whole exocyst complex to the PM [7,34]. Therefore, these intracellular structures might also represent coalesced secretory and recycling compartments that contain aberrant exocyst complexes unable to target secretory vesicles to the PM. [34]. Additionally, it was shown that in *exo70a1* mutant the auxin efflux carrier PIN2 accumulates in the expanded endomembrane compartments [10]. Similarly, a SEC14-like protein, Patellin 3 (PATL3), accumulates in the intracellular structures when expressed in *exo70a1* mutant [35]. This indicates high importance of EXO70A1 in PM targeting of different proteins within a cell. In contrast to the clear secretory function of EXO70A1, the function of EXO70B1 is far more enigmatic. EXO70B1 was shown to play role in autophagic transport to the vacuole [20], trafficking of the FLS2 to the plasma membrane, [36] and was also suggested to be guarded by leucine-rich repeat-containing-like disease-resistance protein TN2 [22]. While anthocyanin accumulation defects are typical for the autophagic mutants [37], reduction in anthocyanin content in *exo70b1* mutant may also be explained by indirect influence of the immune responses.

Even though EXO70B1 and EXO70B2 exhibited the same lipid-binding capacity [38], it is possible that functional divergence of these two paralogs lies within different protein interacting partners such as TN2 [22,39]. In this context it is biologically relevant to stress that the split of EXO70B function into two functionally distinct isoforms seems to be specific for representatives of the Brassicaceae family. Most Angiosperm families have just one EXO70B paralog (e.g., Solanaceae) or more independently duplicated EXO70B paralogs [6].

Several pairs of closely related paralogs within the EXO70.2 subfamily seem to have diversified their functions. For example, two closely related paralogs of the EXO70H clade, EXO70H3, and EXO70H4, share 52% sequence identity and most probably function in different processes. EXO70H4 was the only EXO70 paralog able to complement the trichome callose deposition defect of *exo70h4* mutant indicating a unique function of EXO70H4 in trichomes [32]. EXO70C1 and EXO70C2 paralogs share 38% sequence identity and exhibit a limited degree of functional redundancy in pollen. The *exo70c2* mutant showed a drastic pollen-specific transmission defect due to aberrant pollen tube growth, unlike *exo70c1* mutant. However, disruption of both genes at the same time caused a complete pollen-specific transmission defect [25]. In contrast, duplications of *SEC5* and *SEC10* genes were recent and occurred independently in different plant lineages during evolution [5]. For example, the most recently duplicated exocyst subunit isoforms, SEC10a and SEC10b, share 99% sequence identity at the protein level and a mutation in either of them did not show any phenotypic defect [40]. Similarly, *SEC5a* and *SEC5b* share 80% sequence identity and have redundant functions in the pollen tube growth [41].

## 4. Materials and Methods

### 4.1. Plant Material and Growth Conditions

The Arabidopsis *exo70a1-2* (SALK_135462; [9]) and *exo70b1-2* (GK-156G02; [20]) lines were described previously. Genotypes of individual plants were analyzed by PCR genotyping (for primers see Supplemental Table S1). Seeds were surface-sterilized (70% ethanol for 3 min, 20% commercial bleach for 5 min, rinsed four times with sterile distilled water) and stratified for 2 days at 4 °C. Seeds were germinated on vertical MS agar plates (one-half-strength Murashige and Skoog medium (Duchefa Biochemie, Amsterdam, Netherlands) supplemented with 1% sucrose, vitamin mixture, and 1% plant agar (Duchefa BiochemieAmsterdam, Netherlands) at 22 °C under long-day conditions (16 h light/8 h dark cycles). Seedlings were transferred to turf pellets (Jiffy Products International, Stange, Norway) after 8 days and grown under the same growth conditions.

### 4.2. Cloning of EXO70 Genes

All constructs were prepared using the MultiSite Gateway^®^ (Invitrogen, Waltham, MA, USA). For preparation of expression constructs, the *EXO70A1* and *EXO70B1* promoters (1 kb upstream from the start codon) were subcloned into pDONR P4-P1r, the *GFP* gene in pEN-L1-F-L2 was obtained from VIB in Ghent [42], coding sequences of *EXO70A1* (At5g03540), *EXO70A2* (At5g52340), *EXO70B1* (At5g58430), *EXO70B2* (At1g07000), *EXO70C1* (At5g13150), *EXO70D2* (At1g54090), *EXO70F1* (At5g50380), *EXO70H1* (At3g55150), and *EXO70H7* (At5g59730) with stop codons were subcloned into pDONR P2R-P3 using the Gateway BP clonase mix (Invitrogen, Waltham, MA, USA). A promoter, *GFP*, and an *EXO70* gene were then recombined together into the pB7m34GW destination vector [42] using the Gateway LR clonase mix (Invitrogen, Waltham, MA, USA). The insertions in pDONR vectors were sequenced using M13 primers. The final constructs were sequenced using M13 primers and two outward GFP primers (Supplemental Table S1).

### 4.3. Complementation Assays

Expression constructs with the *EXO70A1* or *EXO70B1* promoters were then introduced to *exo70a1* heterozygous mutants or *exo70b1* homozygous mutants, respectively, using the *Agrobacterium tumefaciens*-mediated floral dip method [43]. Transformants were selected by spraying with glufosinate solution (BASTA; 150 mg∙L^−1^) onto 7-days-old seedlings grown in soil. Complementation assays were performed in plants segregating in the T2 generation, using at least two independent transformed lines for each construct. At least 10 plants were evaluated for each line.

### 4.4. Anthocyanin Induction and Assessment

Plants were grown for one month under short day conditions (8 h light/16 h dark) and transferred to the long day conditions (16 h light/8 h dark) for another month under standard illumination of 100 μM/m^2^/s. Then, rosette leaves were harvested (20 mg and per each sample) and grinded in liquid nitrogen, followed by addition of 2 mL of acidic methanol (1% HCl in 50% MeOH). Samples were centrifuged twice at 14,000 rpm, at room temperature for 5 min. Absorption was measured at 530 nm using a Helios Alpha spectrophotometer (Varian) in 2 mL cuvettes. At least 5 plants (samples) per each genotype were analyzed and each sample contained two technical replicas. The whole experiment was performed in two biological replicates.

### 4.5. Microscopy

The subcellular localization of GFP-tagged EXO70s in roots of 5-day-old or cotyledons of 7-day-old seedlings was performed using a Zeiss LSM 880 confocal laser scanning microscope equipped with C-Apochromat 40×/1.2 WI and 63×/1.4 WI. Samples were illuminated with a 488 nm laser, GFP fluorescence was recorded at 493–535 nm, and chloroplast or FM4-64 fluorescence was recorded at 575–650 nm. For visualization of cellular membranes, seedlings were labelled by FM4-64 (Invitrogen, http://www.invitrogen.com, accessed on 25 April 2021) at a final concentration of 5 μM 30 min before observation.

## 5. Conclusions

EXO70A1 was documented to be involved in the canonical function of the exocyst complex in regulation of polarized exocytosis, while EXO70B1 acquired a diverged and specialized function. Our parallel cross-complementation assays revealed that out of the EXO70 isoforms tested, only EXO70A2 provided a redundant function to EXO70A1 in the Arabidopsis sporophyte. The EXO70B1 function seems to be unique, because no other EXO70 isoform could complement the EXO70B1 disruption. Although functions of several EXO70 isoforms remain to be uncovered, these results clearly document functional specialization within the EXO70 gene family in land plants.

## Data Availability

Data is contained within the article and supplementary material.

## Supplementary Materials

The following are available online at https://www.mdpi.com/article/10.3390/ijms22147595/s1.

## Abbreviations

GFP	green fluorescent protein
PM	plasma membrane
WT	wild type
PIP2	phosphatidylinositol 4,5-bisphosphate
CDS	coding sequence
