# Supplementary material for: Functional Specialization within the EXO70 Gene Family in Arabidopsis
Source: Int J Mol Sci. 2021 Jul 15;22(14):7595. doi: 10.3390/ijms22147595 (PMC8303320; doi:10.3390/ijms22147595)
Supplement: Supplementary file 1 [file ijms-22-07595-s001.zip › ijms-1288487-supplementary.pdf]

Supplementary Materials

Functional specialization within the EXO70 gene family in Arabidopsis

Vedrana Marković, Ivan Kulich, Viktor Žárský

Supplemental Figures

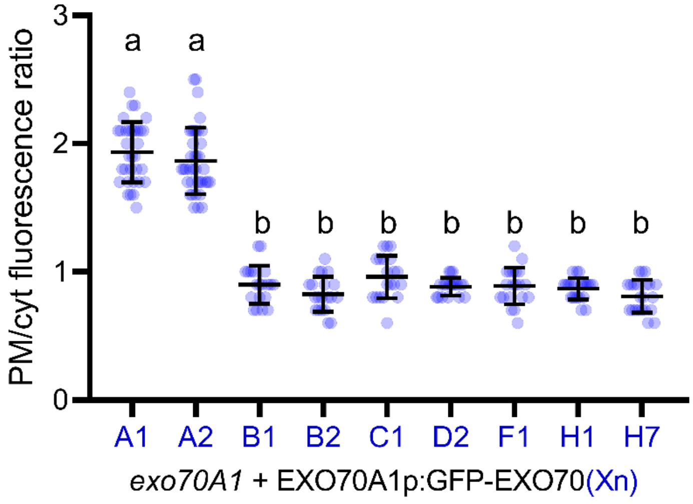

Supplemental Figure S1. EXO70A1 and EXO70A2 localize at the PM in *exo70a1* root epidermal cells unlike other EXO70 isoforms; PM association of GFP-tagged EXO70 isoforms expressed under the *EXO70A1* promoter in the *exo70a1* mutant background (related to Figure 3); the PM association was calculated as a ratio between the average GFP fluorescence intensity at the outer lateral PM and in the adjacent cortical cytoplasm; each dataset represents measurements from at least 25 root cells; letters denote statistically different groups calculated by one-way ANOVA with post-hoc Tukey's honest significant difference test; $P < 0.01$.

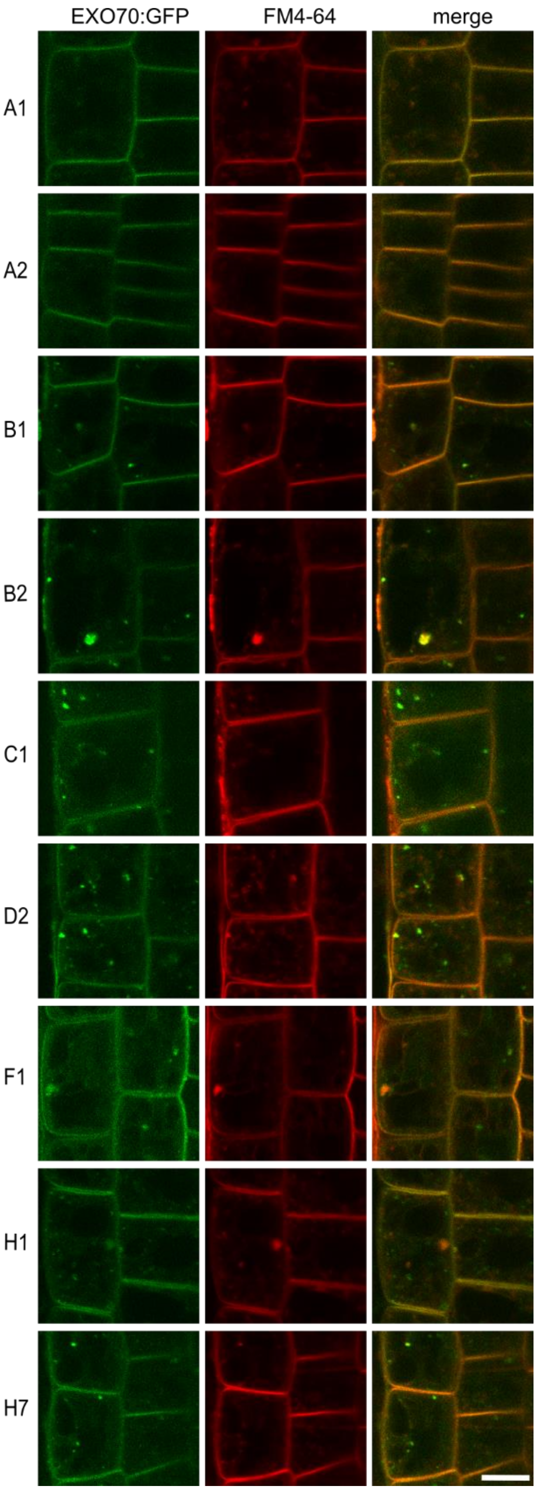

Supplemental Figure S2. Aberrant structures containing EXO70 isoforms in root epidermal cells of *exo70a1* are coalesced endomembrane compartments; seedlings of the *exo70a1* mutant expressing different EXO70 isoforms under the EXO70A1 promoter were stained by FM4-64 dye 30 min before imaging; unlike EXO70A1:GFP and EXO70A2:GFP, other EXO70 paralogs accumulated in expanded endomembrane compartments as documented by colocalization with the FM4-64 staining; scale bars = 10 μm.

Supplemental Table S1. List of primers used in this study**.**

Plant genotyping	
exo70a1-2 LP	TCCATGGACACAAATTTCATG
exo70a1-2 RP	TCTACTGGCATTTTCCCAATGT
LBb1.3	ATTTTGCCGATTTCGGAAC
exo70b1-2 LP	CGTGGCAGGAGTTAGAAGATG
exo70b1-2 RP	TTGTCTGCGTTTTTCCCTATG
Gabi LB 08409	ATATTGACCATCATACTCATTGC
Cloning of EXO70A1p:GFP:EXO70 and EXO70B1p:GFP:EXO70	
A1_prom_fw	ATTGTAAAAAGGGAATGAGCAT
A1_prom_rev	AAAATAACGAATAATCTTTCTGAGTTGA
B1_prom_fw	TAGAAAAGTTGAATGCGGTAGAAGAGAGG
B1_prom_rev	TTTGTACAAACTTGGATTGAAACAGATGTGGAACC
A1_FW	CTTGTACAAAGTGGCTATGGCTGTTGATAGCAGA
A1_RV	GTATAATAAAGTTGTTACCGGCGTGGTTC
B1_FW	CTTGTACAAAGTGGCTATGGCGGAGAATGGT
B1_RV	GTATAATAAAGTTGTCATTTTCTTCCCGTGGTA
A2_FW	GGGGACAGCTTTCTTGTACAAAGTGGCTATGGGGGTGGCTC
A2_RV	GGGGACAACTTTGTATAATAAAGTTGCTTTATCTCTTTGGCTCACTCC
B2_FW	CTTGTACAAAGTGGCTATGGCTGAAGCCGG
B2_RV	GTATAATAAAGTTGTCAACTTGAGCTTTCCTTGA
C1_FW	GGGGACAACTTTGTATAATAAAGTTGCTTTATCTCTTGCGTGCC
C1_RV	GGGGACAGCTTTCTTGTACAAAGTGGCTATGGAGAAATCTGGAAATCAC
D2_FW	CTTGTACAAAGTGGCTATGGCAACACCGGA
D2_RV	GTATAATAAAGTTGTCACTGAGACCGTCTC
F1_FW	GGGGACAGCTTTCTTGTACAAAGTGGCTATGGCCGCAACAAC
F1_RV	GGGGACAACTTTGTATAATAAAGTTGCTTTAACTTTTCCTTCTCGGG
H1_FW	GGGGACAACTTTGTATAATAAAGTTGCTTCAGCCTGAAACACAC
H1_RV	GGGGACAGCTTTCTTGTACAAAGTGGCTATGGCGAAAATGGCG
H7_FW	GGGGACAACTTTGTATAATAAAGTTGCTTCATTCAATGACTACTACGTC
H7_RV	GGGGACAGCTTTCTTGTACAAAGTGGCTATGGGGAAGCATTTATTCC
attB2r adaptor	GGGGACAGCTTTCTTGTACAAAGTGG
attB3 adaptor	GGGGACAACTTTGTATAATAAAGTTG
attB1R adaptor	GGGGACTGCTTTTTTGTACAAACTTG
attB4 adaptor	GGGGACAACTTTGTATAGAAAAGTTGAA
Sequencing of EXO70A2 constructs in Gateway vectors	
M13_FW	GTAAAACGACGGCCAGT
M13_RV	AACAGCTATGACCAT
GFP_seq_FW	CCACAACGTCTATATCATGG
GFP_seq_RV	ACGCCGTAGGTCAG
